# Using digital technologies to facilitate social inclusion during the COVID‐19 pandemic: Experiences of co‐resident and non‐co‐resident family carers of people with dementia from DETERMIND‐C19

**DOI:** 10.1002/gps.5886

**Published:** 2023-02-13

**Authors:** Ben Hicks, Kate Gridley, Josie Dixon, Kate Baxter, Yvonne Birks, Carmen Colclough, Anomita Karim, Rotem Perach, Elen Moseley, Alice Russell, Harsharon K. Sondh, Bryony Storey, Eva Tipping, Riona Mc Ardle, Paul Donaghy, Margaret Dangoor, Eleanor Miles, Louise Robinson, Jennifer Rusted, Harriet Waine, Katherine Wheatley, Sube Banerjee, DETERMIND team

**Affiliations:** ^1^ Brighton and Sussex Medical School University of Sussex Brighton UK; ^2^ Social Policy Research Unit University of York York UK; ^3^ Care Policy and Evaluation Centre London School of Economics and Political Science London UK; ^4^ School of Psychology University of Sussex York UK; ^5^ South London and Maudsley NHS Foundation Trust London UK; ^6^ Gateshead Health NHS Foundation Trust Gateshead UK; ^7^ Newcastle University Newcastle Upon Tyne UK; ^8^ Faculty of Health University of Plymouth Plymouth UK

**Keywords:** carers, COVID‐19, dementia, digital technology, qualitative, social inclusion

## Abstract

**Background:**

The COVID‐19 pandemic triggered rapid and unprecedented changes in the use of digital technologies to support people's social inclusion. We examined whether and how co‐resident and non‐co‐resident family carers of people with dementia engaged with digital technologies during this period.

**Methods:**

Throughout November 2020‐February 2021, we interviewed 42 family carers of people with dementia from our DETERMIND‐C19 cohort. Preliminary analysis was conducted through Framework analysis, followed by an inductive thematic analysis.

**Findings:**

Digital technologies served as a *Facilitator for social inclusion* by enabling carers to counter the effects of the differing restrictions imposed on them so they could *remain socially connected and form a sense of solidarity, access resources and information, engage in social and cultural activities* and *provide* *support* *and independence in their caring role*. However, these experiences were not universal as carers discussed some C*hallenges for tech inclusion*, which included *preferences for face‐to‐face contact, lack of technological literacy* and issues associated with the *accessibility of the technology.*

**Conclusion:**

Many of the carers engaged with Information and Communication Technologies, and to a lesser extent Assistive Technologies, during the pandemic. Whilst carers experienced different challenges due to where they lived, broadly the use of these devices helped them realise important facets of social inclusion as well as facilitated the support they provided to the person with dementia. However, to reduce the ‘digital divide’ and support the social inclusion of all dementia carers, our findings suggest it is essential that services are attuned to their preferences, needs and technological abilities.

## Key Points

- Many co‐resident and non‐co‐resident carers used Information and Communication Technology and Assistive Technology during the pandemic.
- The digital technology supported them to retain their social inclusion and manage the care of the person with dementia.
- Carers faced different challenges when using the digital technology depending on where they lived and on the severity of the person's dementia that they were supporting.
- Formal services must be attuned to the preferences, needs and technological abilities of carers of people with dementia if they are to address the ‘digital divide’ and support their social inclusion.

## 1 INTRODUCTION

‘Social inclusion’ emphasises the need for people to have the material means as well as the agency and unconditional opportunities to access, participate in, and personally grow from social and cultural experiences and inter‐personal relationships that are valued and meaningful to them.^1^, ^2^, ^3^, ^4^, ^5^ It is widely regarded as a dynamic process that individuals and communities experience over time, in different situations and within their wider experiences of social exclusion.^6^, ^7^, ^8^ Within an increasingly digitised society, having access to digital technologies that enable participation is increasingly posited as a human right and an integral component of social inclusion.^9^, ^10^

Over recent years, an increasing range of technologies have emerged within the dementia care arena and scholars have highlighted ways they can support the social inclusion of people with dementia and their carers.^11^, ^12^, ^13^ Digital Assistive Technology (AT) such as medication aids and locator devices can overcome some of the cognitive and physical challenges of dementia, thereby providing people with dementia and their carers with a greater sense of independence.^12^, ^14^ Information and Communication Technology (ICT) such as telecare and virtual social platforms can provide a means for people to socially connect with informal support networks and dementia care services when face‐to‐face engagement may not be possible.^15^, ^16^ Finally, off‐the‐shelf digital Gaming Technology (GT), which supports interactive electronic games such as tablets or motion sensor exergaming devices (e.g. Nintendo Wii and Microsoft Kinect) can provide opportunities for people with dementia and their carers to participate in new leisure experiences that are potentially stimulating and enjoyable^11^, ^17^ as well as enable them to master new and sometimes complex skills, increasing their perceived self‐confidence.^18^, ^19^

Despite these benefits, people with dementia and their carers face multiple barriers to engaging with these technologies, thereby inhibiting their abilities and rights to participate fully in society. Challenges can be attributed to generational (e.g. older people without the knowledge, skills or confidence to use technology) or geographical (e.g. rural‐dwelling with limited/intermittent access to the Internet) determinants that may be prevalent in older carers and so contribute to their digital divide.^20^ Other difficulties include those directly linked to the biopsychosocial challenges of dementia. Many digital technologies have not been designed with people with dementia in mind resulting in interfaces that may be cognitively or physically demanding for this group.^12^, ^13^, ^16^ Other research suggests the cognitive efforts required to use social media can act as a barrier to engagement for some people with dementia, and that stigmatising attitudes and negative language surrounding the condition can be common on these platforms.^21^ Consequently, carers are likely to be required to support the engagement of the person with dementia, further adding to their strain and detrimentally impacting on their independence. These challenges may be difficult to overcome particularly if carers lack the knowledge and skills to select and, where necessary, adapt technologies to ensure they are inclusive of people with dementia.^22^

In late 2019, a new coronavirus (COVID‐19) emerged causing global disruption and stringent restrictions on social contact, activities and service provision, resulting in adverse impacts particularly on the well‐being, burden and support capabilities of family carers of people with dementia.^23^, ^24^, ^25^, ^26^ It also triggered rapid and unprecedented changes in the use of digital technologies throughout society^27^ as these became essential for many people to sustain social connection.^28^ Given the aforementioned challenges that people with dementia and their carers may encounter when engaging with digital technologies, existing inequalities and the ‘digital divide’ may have been exacerbated, detrimentally impacting social inclusion for those affected by the condition.^29^ To promote a future socially‐inclusive dementia care agenda, it is of value to explore whether and how people with dementia and their carers used digital technologies during the pandemic, examining both the benefits and challenges experienced. This could provide important insights to inform policy and practice that seeks to support social inclusion.

Currently, qualitative research that specifically addresses this topic is scarce. The majority of studies examine, more broadly, the experiences of people with dementia and their carers during the pandemic and often note an increase in their use of digital technologies to engage with formal health and social care services.^30^, ^31^, ^32^, ^33^ Although Giebel et al^34^ found that those unpaid carers and people with dementia that accessed digital support during this period reported these services to be of poorer quality and less effective than in‐person contact. One UK study that explored the use of ICT by people with dementia during the pandemic found they used these mediums to facilitate social connection, engage in hobbies and interests, and assist in activities of daily living; although people with dementia also encountered accessibility challenges due to cognitive fatigue and usability issues with the technology.^35^ However, the authors' noted that participants tended to be younger with mild‐to‐moderate dementia, occupied privileged socio‐economic positions and were already using digital technology pre‐pandemic. Consequently, it is unknown whether the findings can be extrapolated to older people from more diverse backgrounds, and who may be less accustomed to these devices. Another study qualitatively examined the use of digital technologies by people with memory concerns and their predominantly co‐resident carers (90% of the sample) in the United States.^36^ They found that carers used technology to remain socially connected, to reduce boredom by streaming music and films or attending online classes, and to enable periods of respite and independence through the use of Global Positioning System (GPS) technologies, which helped to ensure the person with dementia was safe whilst outside alone. The authors noted that carers often had to be present when the person with dementia engaged with a technology, and if the family carer lacked computer literacy or the person's dementia was too severe, then this could add to carers' stress and the person with dementia's confusion, and so provide challenges for the digital inclusion of both.

Adopting a similar qualitative approach, we aim to build on these preliminary findings by exploring, in‐depth, carers' experiences of using digital technologies during the pandemic as a means to sustain their social inclusion as well as to facilitate the support they provide to the person with dementia they care for. Our large and diverse sample of participants, purposively selected from the DETERMIND study,^37^ enabled us to consider the experiences of people from a wide range of socio‐demographic backgrounds who were supporting people with varying severities of dementia. In particular, our design enabled us to draw comparisons between the experiences of carers who live with the person they care for (hereafter ‘co‐resident carers’) and those who care for someone living elsewhere (hereafter ‘non‐co‐resident carers’). This is important, since it is likely they encountered different challenges throughout the pandemic, potentially affecting the digital technologies they chose to adopt (if indeed they did) and the purpose for which they were used.

## 2 METHODS

### 2.1 Research approach

This paper reports on one key theme that was elicited from broad‐ranging interviews with carers' during the COVID‐19 pandemic; namely ‘the use of digital technology during the pandemic.’ Data were collected as part of DETERMIND‐C19, which is situated within the wider DETERMIND programme. Further details of these studies are provided in Supplementary Table S1.

Ethical approvals for the DETERMIND and DETERMIND‐C19 studies were obtained from the UK Health Research Authority Brighton and Sussex Research Ethics Committee [REC 19/LO/0528. IRAS 261263].

### 2.2 Participant recruitment

A purposive sample of 68 of the 114 carers participating in the DETERMIND‐C19 study were approached to discuss their experiences of the pandemic. They were selected to ensure we had a good range of people in different socio‐economic situations. A total of 42 carers (20 co‐resident and 22 non‐co‐resident carers) agreed to be interviewed. Consent was obtained verbally over the phone and recorded on a digital consent form. Participation was voluntary with no monetary incentives provided. Demographic characteristics of the carers are presented in Table 1.

**TABLE 1:** Sampling table of carer characteristics.

Characteristics	Co‐resident	Non‐co‐resident
	(*N* = 20)	(*N* = 22)
	*n*	*n*
Age		
20–34	1	1
35–49	1	2
50–64	2	11
65–79	9	8
80 and above	7	0
Gender		
Female	14	18
Male	6	4
Relationship to person with dementia		
Spouse	19	0
Daughter	0	15
Son	0	4
Granddaughter	0	2
Grandson	1	0
Sister	0	1
Area		
Urban: Major	9	13
Urban: Sparse	6	5
Rural: Town and fringe	4	1
Rural: Village	0	2
Rural: Isolated dwellings	1	0
Missing data	0	1
Type of dementia		
Alzheimer's disease	9	16
Vascular dementia	4	2
Mixed	3	2
Other/Unknown	4	2
Employment status in pandemic		
Full‐time carer	1	3
Employed	0	3
Self‐employed	1	0
Volunteer	1	1
Retired	13	7
Unemployed	1	5
Made redundant	2	1
Furloughed	0	1
Reduced working hours	1	0
Change in employment	0	1
Accommodation status		
Homeowner	17	15
Council rented	1	3
Housing association rented	0	3
Other	2	1
IMD^a^ quintile		
1 (most deprived)	2	5
2	2	3
3	7	4
4	5	3
5 (most affluent)	4	5
Missing data	0	2
Ethnicity		
Asian (unspecified)	0	1
Caribbean	0	1
Indian	1	0
White/Black African	1	0
White/Black Caribbean	0	1
White British	18	19
Engaged with digital tech in pandemic		
Yes	18	20
No	2	2

^a^ Indices of Multiple Deprivation.

### 2.3 Interviews

Open, in‐depth ‘responsive’ interviews enabled a flexible style of interviewing with questions evolving in response to the interviewee's discussion, thereby enabling a deeper exploration into the experience and knowledge of each interviewee.^38^ Therefore, although we had an interview schedule, this was used flexibly and was focussed around three broad areas: (i) what were the participants' experiences and perceptions of the pandemic currently, (ii) how did these compare to the start of the lock‐down (March 2020), and (iii) what were their thoughts on how to move forward through the pandemic. Within each of these three broad areas we used questions and prompts to elicit carers' experiences of the various aspects of their social inclusion that were most important for them. These included socialising and engaging with family, friends and the community, accessing services, and supporting the person with dementia.

Interviews took place between November 2020‐February 2021 (during the second and third UK lock‐down where partial restrictions were in place) and were conducted via telephone or online digital platform (e.g. Zoom) by three experienced qualitative researchers (BH, JD, KG). The interviews ranged in duration from 31 to 101 min excluding consent processes. All interviews were audio recorded and professionally transcribed, anonymised and uploaded into Excel to manage the data analysis process. Pseudonyms preserved individuals' identities.

### 2.4 Data analysis

This took place in two stages. Preliminary analysis of the whole dataset was conducted using Framework analysis.^39^ This preliminary stage was undertaken as a means to reduce the initially large dataset into something that was more easily manageable. Subsequently, an inductive thematic analysis of the data was conducted to examine specifically carers' use of digital technologies. This followed the 6‐stage analysis process as outlined by Braun and Clarke^40^ which involved developing initial codes at a latent and semantic level and then constructing these into themes and sub‐themes through the development of mind‐maps and discussions amongst the wider research team. Table 2 provides a more detailed overview of the two‐stage analysis process.

**TABLE 2:** Two stage analysis process.

Stage 1:	Stage 2:
Framework (Ritchie et al., 2013)	Braun and Clarke (2006) six phase thematic approach
An initial thematic framework structure was developed by the researchers who led the interviews (BH, KG and JD). This was based on the topic guide, familiarisation with the interviews and the charting of several transcripts. Separate frameworks for co‐resident and non‐co‐resident carers were constructed and discussed with a multi‐site team of 13 researchers and final refinements undertaken. Following training, all researchers charted the remaining transcripts into the frameworks and included their own analytic notes. The completed frameworks were shared so the team could familiarise themselves with the data and other team members' notes. Following this, pairs of researchers were tasked with developing broad themes within different areas of the frameworks. A meeting was convened to discuss the emerging themes. At this point it was evident that the use of digital technologies was a prevalent theme throughout carers' accounts of the pandemic and featured in most aspects of the frameworks, although co‐resident and non‐co‐resident carers sometimes used these technologies for different purposes. Therefore, in a second stage of analysis, a more detailed inductive analysis of this sought to understand what and how digital technologies were being used by resident and non‐resident carers.	1. Data familiarisation The transcripts were read and re‐read by BH to familiarise himself with the data. 2. Initial codes developed Data were coded individually at three levels: Individual transcripts were read line by line to identify key messages into codes. This occurred at a semantic and latent level. Definitions were drawn up for each code by BH and these were discussed among the research team. 3. Themes searched Relevant codes were collated into potential themes through discussions between the research team. Codes that were not deemed relevant were recorded as miscellaneous. 4. Themes reviewed Thematic mind‐maps were developed to better understand how the themes sat together and to construct higher level themes. 5. Themes defined and named Themes were reviewed by the wider DETERMIND‐C19 research team and then defined and named. Miscellaneous codes were re‐visited to examine whether they sat within the wider findings. A cross case analysis was undertaken to explore any differences in themes between participants based on their socio‐demographic characteristics. 6. Write‐up The most appropriate participant quotes illustrating the key points pertinent to the research were selected for the final manuscript. The inductive themes and codes were then considered through the lens of social inclusion.

## 3 FINDINGS

Across our sample, the majority of carers, irrespective of their sociodemographic background, reported an increase in their use of digital technologies during the pandemic. Primarily these were Information and Communication Technological (ICT) devices although in some instances carers discussed incorporating AT to enable them to better support the person with dementia. Our findings suggested that these technologies could serve as a *Facilitator of social inclusion (Theme 1),* which included enabling co‐resident and non‐co‐resident carers to *remain socially connected and form a sense of solidarity, access resources and information, engage in social and cultural activities* and *provide* *support* *and independence in their caring role*. However, carers also discussed C*hallenges for tech inclusion (Theme 2)*, which included *preferences for face‐to‐face contact, lack of technological literacy* and issues associated with the *accessibility of the technology.* These themes and their sub‐themes are discussed below. Quotes have been anonymised and information provided pertaining to carers' residential status, sex and age.

### 3.1 Theme 1: Facilitator of social inclusion

Both co‐resident and non‐co‐resident carers discussed using multiple forms of digital technologies during the pandemic. These enabled them to retain important facets of their social inclusion despite the lock‐down restrictions.

#### 3.1.1 Sub‐theme 1: Social connection and solidarity

The majority of carers discussed incorporating a range of ICT, including virtual communication platforms and social media, into their lock‐down routines to enable them to remain socially connected with family, friends and the local community within these *“challenging circumstances.”* Co‐resident carers reported sitting alongside the person with dementia and using virtual technologies such as Zoom or Facebook Portal to regularly call family and friends. They welcomed the ability to be able to sit together and connect with friends and family from all over the world on the same screen and *“to see someone's face and know they're ok*”. There was a sense that these technologies enabled a greater sense of social connection than could have been achieved over the telephone, and were easier to facilitate access together, and this was beneficial for carers' psychological well‐being and that of the person with dementia. For instance, one co‐resident carer positively discussed sitting with the person with dementia to call a family member in a care home and participate in their activities.

The care home have put on a few Zoom parties. We sit here and have mince pies and a glass of wine, or whatever, or a cup of tea, and join in with the carols if we want to. (co‐resident, female, 77)

Another spoke of how she and her husband with dementia used Facebook Portal to interact with their grand‐daughter with learning difficulties. The carer discussed how being able to see her face and engage in activities with her was something that brought them joy and this was beneficial for their well‐being and relationship.

Non‐co‐resident carers also discussed setting up these virtual platforms to facilitate social connection with their wider family and the person with dementia, which was welcomed by all parties as it provided a means to *“see them rather than just listen.”* One carer, who regularly contacted her mother in a care home through Facebook Portal during the pandemic, highlighted how the device was able to facilitate their social connection; ensuring they could see each other and have private and meaningful conversations that they may not have wished to speak about in front of the care staff. This was beneficial for the well‐being of both of them.

It’s the first time that she really used it… the portal is dead simple, literally it’s got a photograph of people… she doesn’t have to think about numbers or anything. She doesn’t need staff to help, meaning we can have a fully private conversation… she can actually see us so it’s a big improvement (compared to the phone)… I feel she is able to communicate better if she is able to see me doing something… it gives her something to focus on. Her deterioration seemed to plateau once we started to use the video calls. (non‐co‐resident, female, 63)

The carer also discussed how she sent digital photos to the Portal to provide her mother with a stimulus to begin conversations with care staff. This demonstrates the multiple ways non‐co‐resident carers could use digital technology to facilitate social connection for themselves and the person with dementia.

Given restrictions on face‐to‐face meetings both co‐resident and non‐co‐resident carers discussed using virtual platforms to contact counsellors or peer support groups where they could access emotional support within a *“safe environment”*. One non‐co‐resident carer highlighted the benefits of attending a virtual carer support group that was run by an Admiral Nurse during the lock‐down. She discussed the importance of developing a sense of connection and solidarity with people who did not know her or her mother during these difficult times. Furthermore, the format of the meeting also enabled the Admiral Nurse to observe her body language and consequently call her afterwards to check on her well‐being. This is something that would not have been possible if the meeting was conducted over the telephone.

It’s good to connect with a community of people…That day I was feeling absolutely terrible at what was going on with the carers and she (Admiral Nurse) messaged me in the Zoom and said ‘everything ok?’ She called me afterwards…and was able to connect me with new carers. (non‐co‐resident, female, 58)

Some carers reported using other ICT such as Whatsapp for more regular or daily correspondences with family and friends. One non‐co‐resident carer discussed setting up a family Whatsapp group with the person with dementia included, through which they exchanged daily messages and jovial emojis to ensure on‐going social connection without needing to verbally articulate their thoughts. Another non‐co‐resident carer discussed joining local Facebook groups as a way to develop a sense of solidarity with others in her community when face‐to‐face interactions were restricted. Although she didn't post on the group chat, she found it a comfort to read and relate to their experiences and through this she felt a sense of connection to them.

It (Facebook group) reinforces that also you’re not the only person going through this, and I know it sound awful, but some people do have it an awful lot worse…it reinforces the fact that it could be so much worse, we’re pretty lucky so far. (non‐co‐resident, female, 60).

Our findings suggest that both co‐resident and non‐co‐resident carers used a range of ICT devices to retain social connection with family and their wider community during the pandemic when face‐to‐face visits were restricted. Through this social connection they were able to develop a sense of solidarity, where they understood and could relate to others' experiences. This was beneficial for their well‐being during difficult periods of the pandemic.

#### 3.1.2 Sub‐theme 2: Access to resources and information

During the early stages of the pandemic, older resident carers in particular reported concerns for their own health and that of the person with dementia whilst younger resident carers were especially mindful of bringing COVID‐19 into the household. Consequently, many of them discussed using online shopping for the first time to avoid visiting shops, particularly during busy times. Predominantly this was for food, but also on occasions for clothes, games, and medication (which still had to be picked up from the pharmacy in most cases). For most, this was a positive experience and many continued to rely on online shopping throughout the pandemic for convenience and to minimise the continued risk of contracting COVID‐19.

It’s absolutely marvellous. I don’t know what I would’ve done without it (online shopping) really. (co‐resident, female, 82)

Non‐co‐resident carers discussed using online shopping to order food and other essentials for the person with dementia. This was particularly beneficial for those that lived a considerable distance away. However, both co‐resident and non‐co‐resident carers reported difficulties, particularly at the start of the pandemic, with obtaining delivery slots and this could result in great periods of stress with carers concerned that the people they cared for might have to go without essentials. Sometimes this was because people with dementia were not, at first, always considered a vulnerable group. As one participant highlighted, this was exacerbated by a lack of dementia‐awareness amongst some shops.

Oh, it was on my mind all the time…The pressure of having the only way my mum was going to get any food was me searching out the delivery slots, is immense. I cannot tell you how hard it was to keep it going…I phoned up Sainsbury’s and Tesco’s and tried to get my mum put on the vulnerable person’s list…when I explained about my mum’s health issues and stuff, told me that my mum was not vulnerable enough…So I just saw red! (non‐co‐resident, female, 60)

Some carers discussed using digital ICT, in addition to TV and radio, to access information to keep them informed about COVID‐19, such as looking up case and death rates for example, as well as current Government guidance and regulations. Although generally this was beneficial, one carer reported they were mindful that this could expose them to misinformation whilst another co‐resident carer felt that the ease of access to information through digital means (iPad) had led the person with dementia to become *“obsessed”* and*“overly worried”* about the pandemic, and managing this added to their stress. Furthermore, some carers spoke of using digital ICT to access information that would better enable them to support the health of the person with dementia. For instance, one participant used the NHS website to manage their mother's urinary tract infection and another to better inform herself on dementia following their mother's recent diagnosis.

I came across a MOOC (online course) on the Age UK website, so I decided to do one on ‘Understanding Dementia’ and another one on ‘Preventing Dementia’ which were very helpful. (non‐co‐resident, female, 58)

With restricted access to formal services during the pandemic, ICT played an important role in providing both co‐resident and non‐co‐resident carers with resources and information that enabled them to better support their own well‐being, as well as that of the person with dementia.

#### 3.1.3 Sub‐theme 3: Social and cultural activities

Although many of the participants reported an increase in their use of the television during the pandemic, which could become *‘a bit boring*, there was evidence that family carers used digital technologies to provide them with mental and physical stimulation, as well as a means of escapism. This was particularly important during adverse weather when they were reluctant to venture outside. For instance, one non‐co‐resident carer discussed doing bingo sessions on Zoom with the person with dementia whilst another spoke of them both regularly playing chess with their grandson using the virtual platform. These collaborative activities provided them with mental stimulation and also a means to connect and enhance their relationships with each other and their wider family.

I don’t think we’ve ever had a laugh and a giggle more than we did…He’s (person with dementia) quite good at looking up videos of cats falling out of trees and he likes to share them with me. So we do watch a lot more silly stuff on the telly, and on the internet. (co‐resident, female, 53)

Carers also discussed engaging with digital technologies on their own as a means to unwind and de‐stress. For instance, one spoke of *‘tracking down’* an Internet radio station that played the music he liked, which he listened to when he needed to relax. Another discussed playing games on her laptop.

I love my games on my laptop, just keep myself occupied and calm…I find it like stress free, it’s my way of relaxing (from caring role), when I can. (non‐co‐resident, female, 36)

Furthermore, carers highlighted how they used digital ICT to continue to virtually connect with activities that had previously been undertaken face‐to‐face. These activities were meaningful to them and afforded them a sense of self‐worth and identity over and above that of being a ‘carer’. These included Church and drama groups as well as Zumba and Slimming World classes. There were other instances of participants joining online groups to engage in new hobbies and activities. As one co‐resident carer highlighted, this provided her with a distraction from the pandemic and her caring role as well as a means to better get to know people in her community, which was something she had not been able to do following their recent move prior to the first lock‐down.

I’ve joined a local history group here, and they have monthly Zoom meetings, and webinars and things. So that’s quite good, so those sorts of outside distractions…I just plan to do things and actually talk to more people, or engage in things that are distracting. (co‐resident, female, 61)

#### 3.1.4 Sub‐theme 4: Support and independence in their caring role

There were some, albeit limited examples of carers incorporating AT during the pandemic as a means to support them in their caring role and so retain a level of independence. For non‐co‐resident carers the technologies could enable them to remotely manage some of the care needs of the person with dementia when face‐to‐face visits were restricted. For instance, one non‐resident carer discussed setting her mother up on Face‐time. As she was also looking after three children during the lock‐down, this reduced some of the pressure on her to be physically present with her mother in the mornings to ensure that she was washed and dressed. This was particularly beneficial given that the home caring service, which used to support her mother with these activities, stopped during the early periods of the pandemic.

I enrolled her on Face‐time…That does make a difference, and if I’m not there, I can ring. I can see what she’s doing, and see what she’s put on, make sure that she’s changed or she’s got her clothes on correctly. (non‐co‐resident, female, 62)

Furthermore, two non‐co‐resident carers discussed using a digital ‘pill carousel’ that provided reminders for the person with dementia to take their medication. These were linked up to a service that would then ring the carer if they had concerns. Again, these were beneficial given the restrictions on face‐to‐face visits and the reduction in home care assistance, which may have previously provided these services.

Co‐resident carers also discussed introducing certain ATs into the home environment to attempt to reduce some of the care burden and so provide them with periods of respite. One spoke of setting up a care alarm to monitor her husband in case he fell. As the COVID‐19 restrictions resulted in them spending a lot of time together, the AT gave her the opportunity to leave the house when restrictions permitted whilst providing her with some reassurance about his well‐being; although she was still apprehensive about leaving him on his own for extended periods of time.

I got him a care call badge he wears if he needed help, he’d press that…I’d be frightened to go out for long though…but I’ve got that sort of cover, although I don’t really go far at all. (co‐resident, female, 83)

### 3.2 Theme 2: Challenges for tech inclusion

Despite carers reporting on the numerous benefits digital technologies offered them throughout the pandemic there were instances where co‐resident and non‐co‐resident carers discussed difficulties introducing them into their care routine. This had important implications for their well‐being during this stressful period.

#### 3.2.1 Sub‐theme 1: Preference for face‐to‐face contact

A common theme was participants' preference for face‐to‐face contact. Many carers, and particularly those who were older, described virtual communication as *“an unsatisfactory medium”* compared to face‐to‐face interactions as they felt the *“quality of the contact”* was poorer. However, generally amongst our carers there was an acknowledgment that for the time‐being these devices were the *“next best thing”* to keep in social contact with their family and friends. On rare occasions, carers discussed how the person with dementia had chosen not to engage with the technology because it did not fulfil the level of connection they desired. Consequently, they were unable to incorporate the digital technologies into their care routine as they would have hoped and so sole responsibility for providing social connection for the person with dementia throughout the pandemic was left to the carer.

At the end of the day (person with dementia) will turn around and say ‘when can I see our son? When can I see the grandchildren.’ Face‐time is not good enough…she really wants face‐to‐face, probably to touch. *(co‐resident, male, 82)*

#### 3.2.2 Sub‐theme 2: Lack of technological literacy

Carers' lack of knowledge of available technologies and/or how to operate them could serve as a barrier for their use during the pandemic. This was most notable for our older participants, who often required the support of others who were more technologically knowledgeable in order to set‐up and use the technologies at the beginning.

It’s a totally alien thing to me (Zoom), but I’m awfully glad of it. One of our daughters set it all up for me (start of the lock‐down), so I just have to follow the instructions and I can manage it. (co‐resident, female, 82)

Lack of knowledge of digital technologies was also most pertinent for ATs and may explain why this type of technology was rarely discussed during the interviews. For instance, one co‐resident carer spoke of wishing there was a video camera application that she could use to monitor her husband. Another expressed her surprise when a social worker discussed the wide range of AT devices available to her. This had only been brought to her attention following her husband's discharge from hospital after catching COVID‐19.

A limited understanding of digital technologies could result in a fear or mistrust towards it, which again would hinder participants' use of the devices. One participant felt that during the pandemic she was receiving a lot of ‘phishing’ emails and online scams from bank services and this had made her very apprehensive. Consequently, she continued to do all her shopping and banking in person, which could be difficult sometimes to fit around her caring responsibilities. However, as reported by another older co‐resident carer, overcoming this initial fear of the technology and mastering the necessary skills provided them with a sense of achievement as well as enhanced their social inclusion during the pandemic.

I’ve mastered the computer…I was always frightened of it to be honest with you, but I’ve got used to it now, and I found it very, very helpful. (co‐resident, female, 82)

#### 3.2.3 Sub‐theme 3: Accessibility of the technology

Although rare, one co‐resident carer residing within a remote town, reported how they were unable to access support groups now they had moved online as the lack of technological infrastructure in the area meant she was unable to receive a phone signal.

On some occasions, although carers were using digital technologies, they had decided against introducing them to the person with dementia as they believed they would be unable to interact with them. Sometimes this decision was made by carers who felt the person's dementia was too advanced and on other occasions carers perceived the person to have limited interest in the technology. As one non‐co‐resident carer discussed, his mother was *“old school”* and so he felt that it would be difficult to move her onto “*modern methods*”. With the right support for carers and people with dementia they may have been able to engage with digital technology more than was attempted and so take advantage of some of the benefits it may have provided them during the pandemic.

Where carers did attempt to support the person they cared for to use digital technologies they occasionally reported difficulties. Interestingly, these instances were not confined to those people with high Clinical Dementia Rating scores. Carers highlighted that some people with dementia had difficulties following conversations on virtual platforms and could find it *“frustrating and a bit difficult seeing many people on the same screen”* (co‐resident, female, 72). Another discussed how the person with dementia found the social conventions of Zoom difficult to comprehend (Q20).

He’s given up using Zoom…he doesn’t understand the business of you’re muted and if you want to join…you have to hold your hand up and the Chairperson will unmute you… He just can’t understand the concept. (co‐resident, female, 77)

Carers also reported other challenges for people with dementia associated with navigating the technology such as knowing which buttons to push or ensuring the screen was correctly positioned. This meant that most of the time carers were required to sit alongside them to support their engagement, which could add to their stress and workload particularly if they were not a resident carer. A non‐co‐resident carer discussed how the introduction of a pill carousel during the pandemic had not worked out as planned and consequently added to their caring responsibilities and sense of strain.

So there’s probably been four or five occasions where I’ve had to go down…One day I went down she’d actually taken the battery out of the carousel…So for some weeks I ended up having to ring her at 8.30 every morning and 6 o’clock every night just before the alarm went off just to ensure she took the pill. (non‐co‐resident, female, 66)

Another discussed how she was required to ring her mum on the phone to *“guide her”* through how to answer her call over virtual platform, which again added time to her caring responsibilities. These experiences meant that some carers were fearful of a move towards a more digital society post‐pandemic as this may result in the long‐term social exclusion of some people with dementia.

I sometimes find everything’s on the internet now…but my mum can’t do that, she can’t look for help, she can’t do anything with computers…and that is a challenge for her. (non‐co‐resident, female, 52)

## 4 DISCUSSION

Through qualitative, in‐depth interviews, we examined how co‐resident and non‐co‐resident carers of people with dementia experienced the pandemic, the challenges they encountered and the sources of support they drew upon. The use of digital technologies was a prominent theme in the majority of our interviews. Adopting a social inclusion lens, we found that many carers, irrespective of their socio‐demographic characteristics, employed ICTs, and to a lesser extent ATs, at the start of the UK lock‐down. Interestingly there were limited discussions around the use of digital gaming devices during this period, which may suggest, as other research has posited, that currently this is something rarely considered by people with dementia and their carers (due to fear, lack of knowledge or belief it is unsuitable for them) or promoted in the dementia care agenda.^26^, ^27^, ^28^, ^29^

The ICTs and ATs used by the carers we interviewed were largely welcomed as they enabled them to achieve important facets of their own social inclusion as well as helped them to provide care and support to the person with dementia, despite pandemic restrictions. Indeed, for some of our older carers, this unanticipated opportunity to learn how to engage with these digital devices contributed to their sense of achievement. Developing new skills for life‐long learning is posited as an important facet for social inclusion as we age.^4^

### 4.1 Social inclusion of co‐resident carers

For co‐resident carers, these digital ICT devices enabled them to sit with the person with dementia and together satisfy their need for social connection with family, friends and neighbours. They also provided a mechanism for carers to engage in social and cultural activities that were integral to their identities and provided them stimulation, a means to relax as well as, in some cases, opportunities to develop their relationship with the person with dementia and find solidarity with other carers during these difficult times. These are all key components for social inclusion.^4^, ^5^, ^6^, ^7^ Carers who resided with the person with dementia could also help facilitate their interactions with these devices and so support their sense of social connection with family, friends and community groups. ICT also enabled co‐resident carers to remotely access vital resources such as food and medication and so reduce the need for them and/or the person with dementia to put themselves at risk of catching COVID‐19 by visiting shops and services. Furthermore, it provided carers access to information that could help keep them informed on the pandemic, and crucially enabled them to access medical and dementia care information when physical access to formal healthcare services was restricted. On the rare occasions that AT was discussed, it was often seen as beneficial for co‐resident carers as it provided them the opportunity to manage the care needs of the person with dementia whilst enabling them periods of respite and independence from their caring role. These benefits were important for their well‐being.

### 4.2 Social inclusion of non‐co‐resident carers

Non‐co‐resident carers benefitted socially in a similar way to co‐resident carers from the use of digital technology during the pandemic as it facilitated connection with family and friends as well as enabled them to develop a sense of solidarity with other carers. However, they were also able to use the devices to satisfy the different care requirements they encountered due to the restrictions imposed during the pandemic. Non‐co‐resident carers used ICT to arrange for food and medication to be delivered to the person with dementia and so negate the need for them to always do this themselves. This was particularly welcomed by carers who lived a great distance from the person with dementia or who were responsible for providing care to other younger family members. In some cases they were also able to use ICT and AT as a means to remotely manage the care of the person with dementia when face‐to‐face visits were restricted or home care services were no longer being used. This included using virtual platforms to call and check on the well‐being of the person with dementia as well as using AT to remotely manage their medication routine. This was particularly important as other research has shown how paid home care help was discontinued during the pandemic because of Governmental restrictions or the decisions made by carers to reduce the risks of infection, further adding to the care burden particularly on non‐co‐resident carers.^32^, ^41^ Whilst there was some evidence that incorporating these devices was successful, it is important to note that there was a heavy reliance on the carer to set up and manage them from afar. As noted in other research,^36^ this could result in additional stress and strain particularly if the interventions did not work out as planned. This will be re‐visited later in the discussion.

Taken together, our findings support other qualitative research that has suggested ICT could be beneficial for the well‐being of carers and people with dementia during the pandemic.^36^, ^42^ They add to this current knowledge base by outlining how ICT, and to some extent AT, were used by both co‐resident and non‐co‐resident carers from multiple socio‐economic backgrounds to enable them to fulfil different aspects of their caring role and so retain aspects of their social inclusion despite the restrictions. Our findings also provide some support for the growing body of literature, emerging prior to the COVID‐19 pandemic, which advocates the use of digital technologies within the dementia care agenda.^21^, ^22^ However, this does come with important caveats as our research also suggested numerous challenges to technological inclusion that were encountered by both co‐resident and non‐co‐resident carers, which could add to their care burden. Whilst some commentators have suggested that the pandemic may have acted as a spring‐board to accelerate the *“necessary digital revolution”* within dementia care,^43^ if these issues are not addressed then this period may only serve to exacerbate existing inequalities and inequities in the post‐diagnostic care pathway and so further socially exclude certain populations affected by dementia.

### 4.3 Supporting technological inclusion for carers of people with dementia

In keeping with other studies,^36^, ^44^ our findings suggest that one important area to address is technology literacy for carers of people with dementia, and particularly those from an older generation who do not have the experience of using the devices or support from younger or more technologically knowledgeable family members.^44^ Over recent years, resources have been developed to publicise the availability of digital devices and to upskill carers to use them as a means to support the independence, safety and well‐being of people with dementia.^45^ However, it may be that carers, as part of their coping styles to manage the unknown, are reluctant to look too far ahead^46^ and so may not engage with these materials until dementia symptoms have progressed and the challenges of caring for the person become more profound. Research suggests that for the successful adoption of ICT and AT, there is a need for discussions early on in the care pathway between people with dementia, their carers and health and social care workers, around introducing digital technology in a supportive, safe and ethical manner.^23^, ^45^ Moving forward, further training to support dementia practitioners to facilitate these discussions in the early months following a diagnosis, rather than at crisis point, may help to raise awareness of the digital technology available, give carers the opportunity and time to learn how to engage with them as well as outline those that will be acceptable to the person with dementia.

Accessibility of the technology was also a barrier to its use among our participants. Some carers chose not to attempt to use ICT or AT with the person with dementia as they perceived they would not have the capabilities or inclination to interact with them. Whilst carers may have valid reasons for believing that some ICT and AT may not meet the needs of the person they care for, there have been improvements in the usability of ICT interfaces and research shows that, with the right support, technology can be a valuable tool for this group.^12^ This provides further evidence for the need to challenge some of the prevailing defeatist attitudes by publicising research and practice that demonstrates people with dementia successfully engaging with these digital devices and so ‘resisting’ the tragedy discourses associated with the condition.^29^, ^32^ Some of the carers who chose to support the person with dementia to use digital technology did report difficulties associated with the cognitive challenges of dementia or poor social practices that would socially exclude them, such as multiple users talking onscreen. This could add to carers' workload and responsibilities, often meaning they had to be physically present with the person with dementia as they engaged with the technology, which exacerbated their sense of strain particularly for non‐co‐resident carers. Therefore, similar to other researchers,^38^ we advocate the need to develop and publicise training that outlines ways to make these digital devices more dementia‐friendly as well as promotes accessible online environments and practices that are socially inclusive of these populations.

Finally, it is important to note, similar to other research,^31^ that whilst carers and the majority of the people with dementia they supported were happy to engage online during the pandemic due to necessity, there was a strong preference for face‐to‐face contact in *‘normal’* times. This finding reaffirms calls from other research for the urgent re‐introduction of community services that can facilitate in‐person social contact.^34^, ^35^ Moving forward, post‐pandemic, it is important that dementia services re‐engage with person‐centred principles of care to strike the right balance between providing convenient and accessible methods for carers to interact via digital means whilst still ensuring they are socially inclusive and able to fulfil people's desires for face‐to‐face contact.

### 4.4 Strengths and limitations

Purposively sampling from our wider DETERMIND cohort enabled us to explore the accounts of a large number of resident and non‐resident carers from a wide variety of socio‐demographic backgrounds who were supporting people with varying degrees of cognitive impairment. Furthermore, as our recruitment procedure did not rely on social media or online dementia networks we were able to examine the accounts of carers who were less technologically knowledgeable to better understand the barriers they encountered.

However, our study has limitations. All carers were already participating within the wider DETERMIND study and so represent a population that may be more inclined to undertake research. As such, caution must be taken when extrapolating the findings as they may not be representative of the wider population. Furthermore, the carers were supporting people with dementia within their first year of diagnosis and so again are unlikely to represent the breadth of the population, particularly those supporting people with more severe dementia. Whilst our sample reported a range of experiences, those having very difficult times due to the pandemic may have felt unable to participate in this aspect of the research and so offer their valuable insights. Our remote interview methods that were necessitated by the pandemic restrictions may have also meant we excluded some carers who were most at risk of digital exclusion. Finally, while we included participants from Black, Asian and minority ethnic groups, our sample did not allow for detailed analysis of the experiences of specific ethnic or cultural groups.

## 5 CONCLUSIONS

The COVID‐19 pandemic triggered rapid and unprecedented changes in the use of digital technologies throughout society and our data showed that many of the co‐resident and non‐co‐resident carers chose to engage with these devices during this period, often through necessity. The technologies ensured the carers could achieve important facets of their social inclusion as well as facilitate care and support for the person with dementia by overcoming the differing challenges imposed on them by the pandemic. However, we must be mindful as we look to learn from the pandemic. Whilst a greater emphasis on digital technologies within dementia care policy directives may bring benefits for carers it may also exacerbate inequalities and the ‘digital divide’ and so further exclude certain carers. To ensure the social inclusion of all, our findings suggest it is essential that dementia services are attuned to carers' preferences, needs and technological abilities and where appropriate provide dementia‐friendly training to enhance tech literacy and knowledge. It is also vital that the dementia‐friendly communities' agenda^9^ extends to the online environment to ensure that practices are inclusive of people with dementia and those who support them.

## CONFLICT OF INTEREST STATEMENT

The authors declare no conflicts of interest.

## ETHICS STATEMENT

Ethics approval for the DETERMIND and DETERMIND‐C19 studies were obtained by the HRA Brighton and Sussex Research Ethics Committee [REC 19/LO/0528. IRAS 261263].

## Supporting information

Supporting Information S1

## Data Availability

Deidentified participant data will be available with investigator support from 9 months after publication of the last DETERMIND‐C19 paper via sube.banerjee@plymouth.ac.uk for researchers whose proposed use of the data has been approved by the DETERMIND Programme Management Board. This is likely to be in early 2023. The study protocol will be available as a supporting document.
