# Supplementary material for: Using digital technologies to facilitate social inclusion during the COVID‐19 pandemic: Experiences of co‐resident and non‐co‐resident family carers of people with dementia from DETERMIND‐C19
Source: Int J Geriatr Psychiatry. 2023 Feb 13;38(2):e5886. doi: 10.1002/gps.5886 (PMC10947510; doi:10.1002/gps.5886)
Supplement: Supplementary file 1 — Supporting Information S1 [file GPS-38-0-s001.docx]

**Appendix 1**

Supplementary Table 1. Overview of DETERMIND and DETERMIND-C19 studies

| **Study** | **Description** |
| --- | --- |
| DETERMIND (DETERMinants of quality of life, care and costs, and consequences of INequalities in people with Dementia and their carers). (Farina et al., 2020) | ***Funder (Duration):*** ESRC/NIHR (January 2019- December 2024)  ***Aim:*** DETERMIND is designed to address critical, fundamental, and as yet unanswered questions about inequalities, outcomes and costs following diagnosis with dementia. Drawing on seven individual workstreams, DETERMIND’s overall aim is to explore and understand inequalities in dementia care and what drives good and bad quality of life, outcomes and costs for people with dementia and their carers following diagnosis.  ***Inclusion criteria:*** To participate in DETERMIND, people were required to have received a clinical diagnosis of any form of dementia within the six months prior to consenting onto the study. People with Mild Cognitive Impairment or subjective memory complaints as well as those with a diagnosis more than six months prior were excluded. People with dementia who lacked capacity could only participate in the study with the approval of an appropriate personal or nominated consultee. An unpaid (family member/friend) or formal (paid worker) carer could participate alongside the person with dementia as long as they knew them well enough to provide information on the services they had accessed and were familiar with their daily lives.  ***Recruitment:*** Participants were recruited from three geographically and socially diverse areas of England (Sussex/ South London/ Newcastle and Gateshead). These sites were chosen to enable exploration of key socio-economic attributes and their impact on the post-diagnostic care pathway, relevant for the aims of DETERMIND’s individual workstreams. The Sussex region serves wealthy areas with high levels of self-funding (Workstream 4), South Asian older populations (Workstream 2 and 3) and the oldest LGBTQ+ population in the UK (Workstream 2 and 3) as well as more economically deprived, rural coastal dwellings (Workstreams 2, 3 and 4). Southeast London includes the inner city and large populations of older black, Caribbean and south Asian communities (Workstream 2). Finally, Newcastle/Northeast England consists of white working class and rural-dwelling older adults (Workstreams 2, 3 and 4).  ***Data collection:*** All assessments were carried out face-to-face within the participants’ homes. These lasted around two hours with breaks for refreshments. |
| DETERMIND-C19 (Impact of COVID-19 on people newly diagnosed with dementia and their family carers) | ***Funder (Duration):*** UKRI/ESRC (June 2020-September 2021)  ***Aim:*** This mixed method study is nested within the wider DETERMIND programme. It was established following the pandemic, which resulted in a pause in DETERMIND recruitment. The project aims to investigate how social restrictions and changes in health and social care services that were introduced during the pandemic are affecting life under lockdown for people with dementia and their carers. It draws on the pre-pandemic DETERMIND sample, which consists of 266 people newly diagnosed with a range of severities of dementia in the months before the COVID-19 lockdown and their carers.    ***Inclusion criteria:*** All carers recruited via DETERMIND were eligible to participate. Given the difficulty of assessing capacity over the telephone, only those people with dementia who had the capacity to provide informed consent at DETERMIND baseline were approached.  ***Recruitment:*** All participants who were eligible were provided written information on the study and then called a week later to establish whether they wished to participate. For people with dementia, capacity was assessed again remotely by the research team and recorded electronically before undertaking the questionnaires. Where possible, discussions were also held with carers to provide further insight into any changes in the capacity of the person with dementia since baseline interviews. Consent for both people with dementia and their carers was taken over the telephone and recorded electronically.  ***Data Collection:*** DETERMIND-C19 interviews were completed by telephone or self-completion hardcopy that were then posted back to the research team. To enhance data quality across the varying methods, all participants received prompt cards with scale responses that they could refer to during the telephone interview with the researcher. If participants completed the measures via post, then detailed explanations of the measures and instructions for completing it were provided. Researchers also called the participants to check-in with them and ascertain whether they required any support to complete the measures. |

**Supplementary Table 2: Illustrative quotes to support the final themes**

| **Higher order theme** | **Sub-theme** | **Supporting quotes** |
| --- | --- | --- |
| Facilitator of social inclusion | Social connection and solidarity | Q1: *“The care home have put on a few Zoom parties. We sit here and have mince pies and a glass of wine, or whatever, or a cup of tea, and join in with the carols if we want to.” (co-resident, female, 77)*  Q2: “So the kids have been able to Face-time her, so she’s been able to see them rather than just listen to them.” (non-co-resident, female, 46)  Q3: “It’s the first time that she really used it properly and regularly (during the pandemic)…the portal is dead simple, literally it’s got a photograph of people…she doesn’t have to think about numbers or anything. She doesn’t need staff to help, meaning we can have a fully private conversation…she can actually see us so it’s a big improvement (compared to the phone)... I feel she is able to communicate better if she is able to see me doing something…it gives her something to focus on. Her deterioration seemed to plateau once we started to use the video calls.” (non-co-resident, female, 63)  *Q4: “It’s good to connect with a community of people. It’s not the same with friends because they don’t know in the same way and everybody I know, knows my mum. She is well known in the area, which is good…sometimes it’s good that it’s total strangers…That day I was feeling absolutely terrible at what was going on with the carers and she (Admiral Nurse) messaged me in the Zoom and said ‘everything ok?’ She called me afterwards…and was able to connected me with new carers.” (non-co-resident, female, 58)*  Q5: *“It (Facebook group) reinforces that also you’re not the only person going through this, and I know it sound awful, but some people do have it an awful lot worse…it reinforces the fact that it could be so much worse, we’re pretty lucky so far.” (non-co-resident, female, 60).* |
|  | Access to resources and information | Q6: *“It’s absolutely marvellous. I don’t know what I would’ve done without it (online shopping) really.” (co-resident, female, 82)*  *Q7: “Oh, it was on my mind all the time. You know…the pressure of having the only way my mum was going to get any food was me searching out the delivery slots, is immense. I cannot tell you how hard it was to keep it going…I phoned up Sainsbury’s and Tesco’s and tried to get my mum put on the vulnerable person’s list…when I explained about my mum’s health issues and stuff, told me that my mum was not vulnerable enough…So I just saw red!... …So I just saw red! I wrote to the Managing Director…I vented all in this letter…and about four or five weeks later I got an email saying I was entitled to a priority booking.” (non-co-resident, female, 60)*  *Q8: “We like to use his (person with dementia) iPad to get the information…We look at the Government website, because there’s so much misinformation, it gets a bit silly. Having said that, you know, if you look at the BBC website and compare it to the Government website, you can see discrepancies. So I try to stick to the Government website.” (co-resident, female, 66)*  Q9: *“I came across a MOOC (online course) on the Age UK website, so I decided to do one on ‘Understanding Dementia’ and another one on ‘Preventing Dementia’ which were very helpful.” (non-co-resident, female, 58)* |
|  | Social and cultural activities | Q10: *“I don’t think we’ve ever had a laugh and a giggle more than we did…I mean I knew there was some very stupid people in the world, but looking on Facebook, there’s an awful lot more than I realised!...He’s (person with dementia) quite good at looking up videos of cats falling out of trees and he likes to share them with me. So we do watch a lot more silly stuff on the telly, and on the internet.” (co-resident, female, 53)*  Q11: *“I love my games on my laptop, just keep myself occupied and calm…I find it like stress free, it’s my way of relaxing (from caring role), when I can.” (non-co-resident, female, 36)*  Q12: *“We go to church virtually every Wednesday, because I actually set up the readers and responders for Wednesdays, because I volunteered to do so…but we’re a fully Zoomed and streamed church now!” (co-resident, male, 82)*  Q13: *“I’m still a member of the local drama group…I was in a Zoom production at Halloween, which was quite fun.” (co-resident, female, 74)*  Q14: *“I’ve joined a local history group here as well, and they have monthly Zoom meetings, and webinars and things. So that’s quite good, so those sorts of outside distractions…I just plan to do things and actually talk to more people, or engage in things that are distracting, that I know are out there now.” (co-resident, female, 61)* |
|  | Support and autonomy in caring role | Q15: *“I enrolled her on Face-time…That does make a difference, and if I’m not there, I can ring. I can see what she’s doing, and see what she’s put on, make sure that she’s changed or she’s got her clothes on correctly.” (non-co-resident, female, 62)*  Q16: *“I got him a care call badge he wears if he needed help, he’d press that…I’d be frightened to go out for long though…but I’ve got that sort of cover, although I don’t really go far at all.” (co-resident, female, 83)* |
| Challenges for Tech Inclusion | Preference for face-to-face contact | Q17: *“At the end of the day (person with dementia) will turn around and say ‘when can I see our son? When can I see the grandchildren.’ Face-time is not good enough…she really wants face-to-face, probably to touch.” (co-resident, male, 82)* |
|  | Lack of technological literacy | Q18: *“It’s a totally alien thing to me (Zoom), but I’m awfully glad of it on a Monday morning. But one of our daughters set it all up for me (start of the lock-down), so I just have to follow the instructions and I can manage it.” (co-resident, female, 82)*  Q19: *“I’ve mastered the computer…I was always frightened of it to be honest with you, but I’ve got used to it now, and I found it very, very helpful.” (co-resident, female, 82)* |
|  | Accessibility of the technology | Q20: *“He’s given up using Zoom…he doesn’t understand the business of you’re muted and if you want to join…you have to hold your hand up and the Chairperson, whoever happens to be in charge, will unmute you, and you can then say what you want. He just can’t understand the concept.” (co-resident, female, 77)*  Q21: *“So there’s probably been four or five occasions where I’ve had to go down…One day I went down she’d actually taken the battery out of the carousel, so it wasn’t working…So for some weeks I ended up having to ring her at 8.30 every morning and 6 o’clock every night just before the alarm went off just to ensure she took the pill.” (non-co-resident, female, 66)*  Q22: *“Sometimes (the person with dementia) can’t work out how to answer it (virtual platform) and struggles with the ‘answer’ and ‘decline’ buttons. So we will call her on the phone and guide her…I use the mobile as back-up.” (non-co-resident, female, 63)*  Q23: *“I sometimes find everything’s on the internet now, well, that’s fab, that’s great…you know you can spend hours on the computer doing things, but my mum can’t do that, she can’t look for help, she can’t do anything with computers…and that is a challenge for her.” (non-co-resident, female, 52)* |
